# Effect of the Diameter and Angulation Design of the Abutment Cementable Portion on Single Crown Retention: An In Vitro Experimental Study

**DOI:** 10.7759/cureus.87370

**Published:** 2025-07-06

**Authors:** Erika Martins Gomes Baltieri, Sidney Watinaga, Marco A Costa Tritto, Antonio Scarano, Felipe Lorusso, Nilton De Bortoli Júnior, Sergio A Gehrke

**Affiliations:** 1 Dentistry, Universidade Paulista (UNIP), São Paulo, BRA; 2 Oral Medicine, University of Chieti-Pescara, Pescara, ITA; 3 Biotechnology, Universidad Catolica de Murcia, Montevideo, URY

**Keywords:** abutments, axial abutment angulation, cemented crowns, dental implants, tensile strength

## Abstract

Objective

This study aimed to evaluate the influence of abutment geometry, specifically axial wall angulation and diameter, on the tensile strength of cemented prosthetic crowns using two types of abutments (Smart and Ideale) with identical heights but different diameters and angulation of the cementable abutment portion.

Materials and methods

Forty implant-abutment (IA) sets with a Morse taper connection were divided into four groups (n = 10): Sm1 (Smart, 3.5 mm), Id1 (Ideale, 3.3 mm), Sm2 (Smart, 4.5 mm), and Id2 (Ideale, 4.5 mm). Metal copings were cast using a nickel-chromium alloy and cemented with zinc phosphate cement under standardized conditions. Tensile testing was performed to determine the force required to remove each crown. Data were analyzed using parametric tests, with significance set at p < 0.05.

Results

All groups passed the Shapiro-Wilk normality test (p > 0.05). The Id2 group showed the highest mean tensile strength (235.1 ± 9.785 Ncm), followed by Sm2 (191.4 ± 8.870 Ncm), Id1 (150.8 ± 7.745 Ncm), and Sm1 (137.0 ± 7.666 Ncm). Statistically significant differences were observed between several group comparisons, indicating that both abutment angulation and diameter influence prosthesis retention.

Conclusion

Abutments with smaller axial angulation (Ideale model) and larger diameters demonstrated superior mechanical retention. These factors should be considered when selecting abutments for cemented implant-supported prostheses to ensure optimal retention and clinical performance.

## Introduction

With the predictability and longevity provided by osseointegration, dental implants have become a consolidated and widely used alternative in modern oral rehabilitation [1]. Associated with the increase in life expectancy and the growing aesthetic and functional demands of patients, this type of treatment has come to occupy a prominent role in dentistry, also made possible by progressive financial access [2].

Oral rehabilitation with implants is considered safe, effective, and indicated for totally or partially edentulous patients, being a reliable alternative to conventional prosthetic methods [3]. However, the long-term success of this therapy depends on a series of biomechanical and clinical factors, among which the appropriate choice of prosthetic components stands out, including the abutments, connections, and type of prosthesis retention [4]. In the context of cemented prostheses, aspects such as the height and diameter of the abutments, the angulation of the axial walls, the type of cement used, and the geometry of the surface play a fundamental role in the retention and mechanical resistance of the restorations [5,6].

The variation in the convergence angle between the axial walls of the abutments can affect the distribution of load forces during use [7], interfering with the tensile strength and retention capacity of the cemented prosthesis [8]. Then, the greater the angulation, the greater the off-axis force that generates more stress and strain in implant components, especially the screw [7]. The more inclined angulation of the walls of the abutments can favor greater passive adaptation and minimize misalignments during cementation, while the straighter angulation of the abutments can offer less resistance in overload situations, but with aesthetic and initial adjustment advantages. Thus, this geometric characteristic is fundamental for evaluating the performance of the abutments under tensile forces, influencing not only retention but also the durability and longevity of prosthetic rehabilitation.

The literature shows that the retention of cemented metal copings is influenced by the morphological characteristics of the abutments, such as height and convergence angle, as well as by the type of cement used [9,10]. Although screw-retained prostheses are easier to remove and maintain, cemented prostheses offer advantages in terms of aesthetics, seating passivity, and resistance to ceramic fracture, as long as they are carefully planned [11,12].

Therefore, this study aims to compare, through mechanical tensile testing, the retention performance of two types of prosthetic abutments, both with a height of 4 mm, but differing in the diameter (3.3 mm, 3.5 mm, and 4.5 mm) and convergence angle of the cementable portion. The goal is to support the clinical selection of abutments that provide optimized retention and long-term functional predictability.

## Materials and methods

Materials

**Figure 1:**
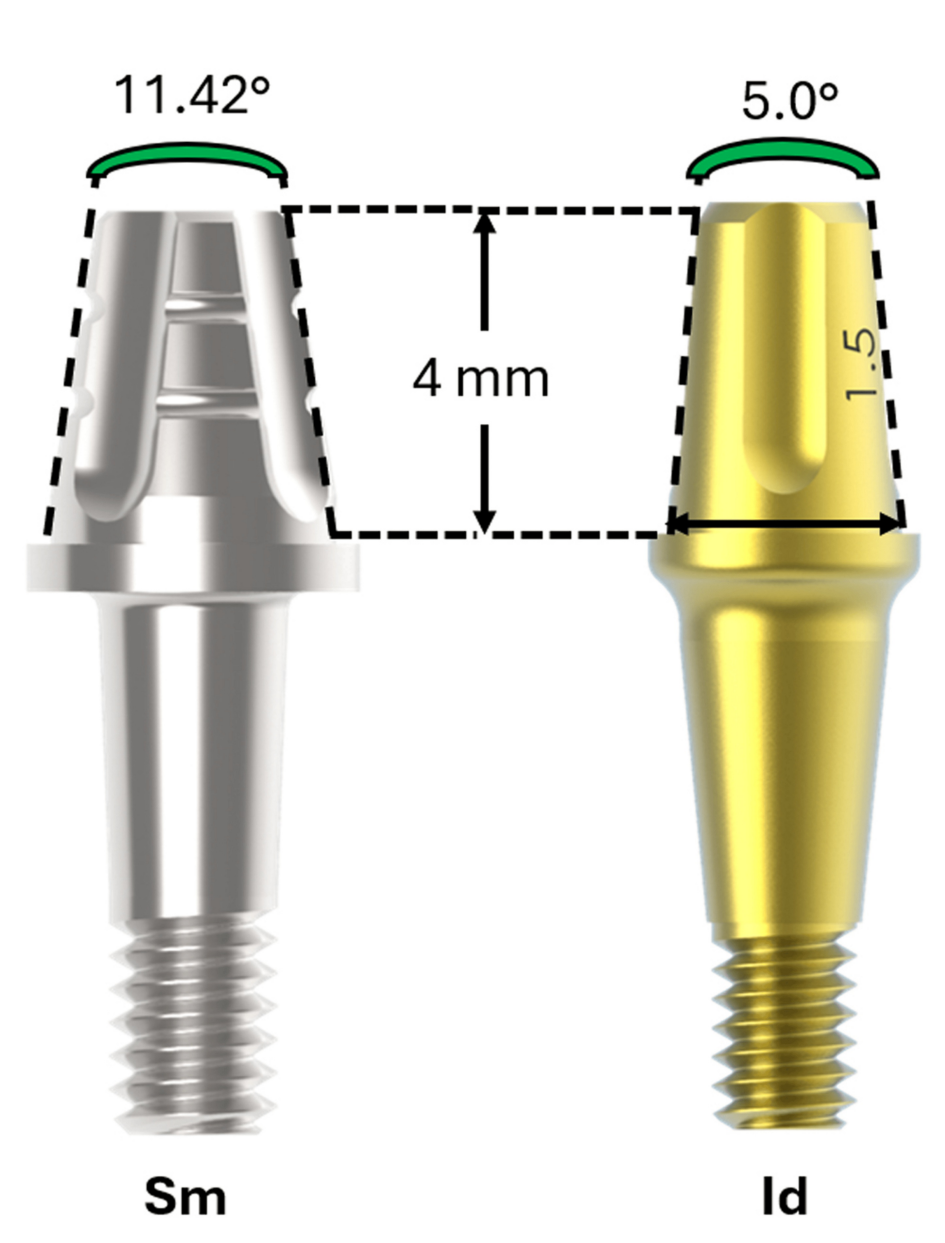
The main differences between the abutments tested Sm = Smart model; Id = Ideale model Source: The Authors

In the present study, straight abutments with two different angles were compared in the cementation portion of the crown: Abutment Smart (Sm), with an angle of 11.42°; and Abutment Ideale (Id) with an angle of 5°. Figure 1 shows the main differences between the abutments tested.

Forty implants and abutments (IA) sets with a Morse cone connection were divided into four experimental groups, according to Table 1.

**Table 1:** Description of the groups and abutment characteristics

Group	n	Abutment	Diameter (mm)	Height (mm)
Id1	10	Ideale	3.3	4.0
Sm1	10	Smart	3.5	4.0
Id2	10	Ideale	4.5	4.0
Sm2	10	Smart	4.5	4.0

**Figure 2:**
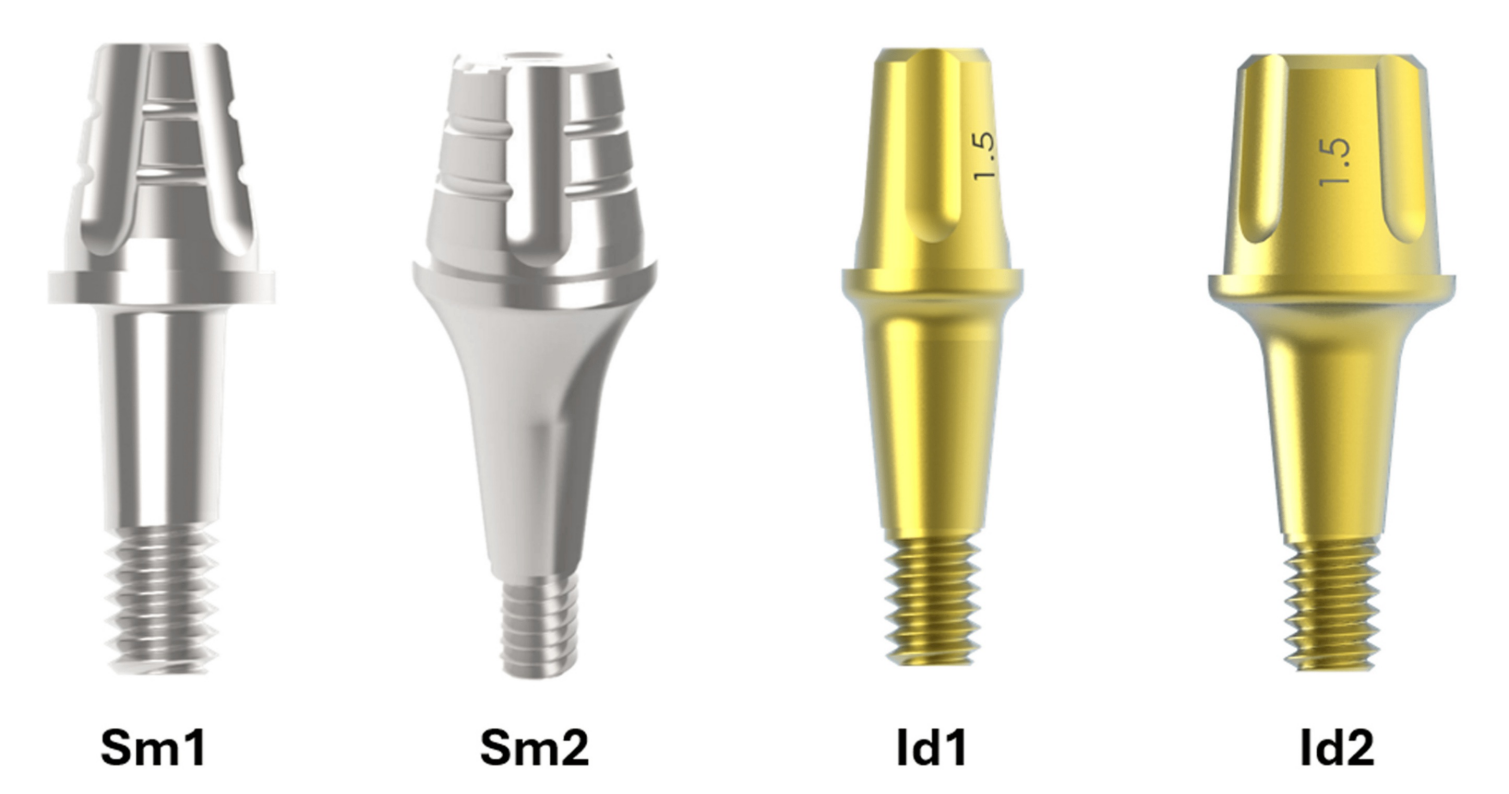
All the solid abutments used Sm = Smart model; Id = Ideale model Source: The Authors

The Smart and Ideale abutment models were selected due to their anatomical characteristics and compatibility with the implants used, both with a height of 4 mm in the cementable portion and variable diameters. The height of the cementable portion of the abutment was selected because it is the most critical among the existing models for the retention of cemented crowns. All abutments and implants used are manufactured and marketed by Implacil De Bortoli (São Paulo, Brazil). The abutments were manufactured in F67 grade IV titanium, according to the manufacturer's specifications. Furthermore, all the abutments used are single-body (solid abutments), as shown in Figure 2.

Metallic copings preparation

**Figure 3:**
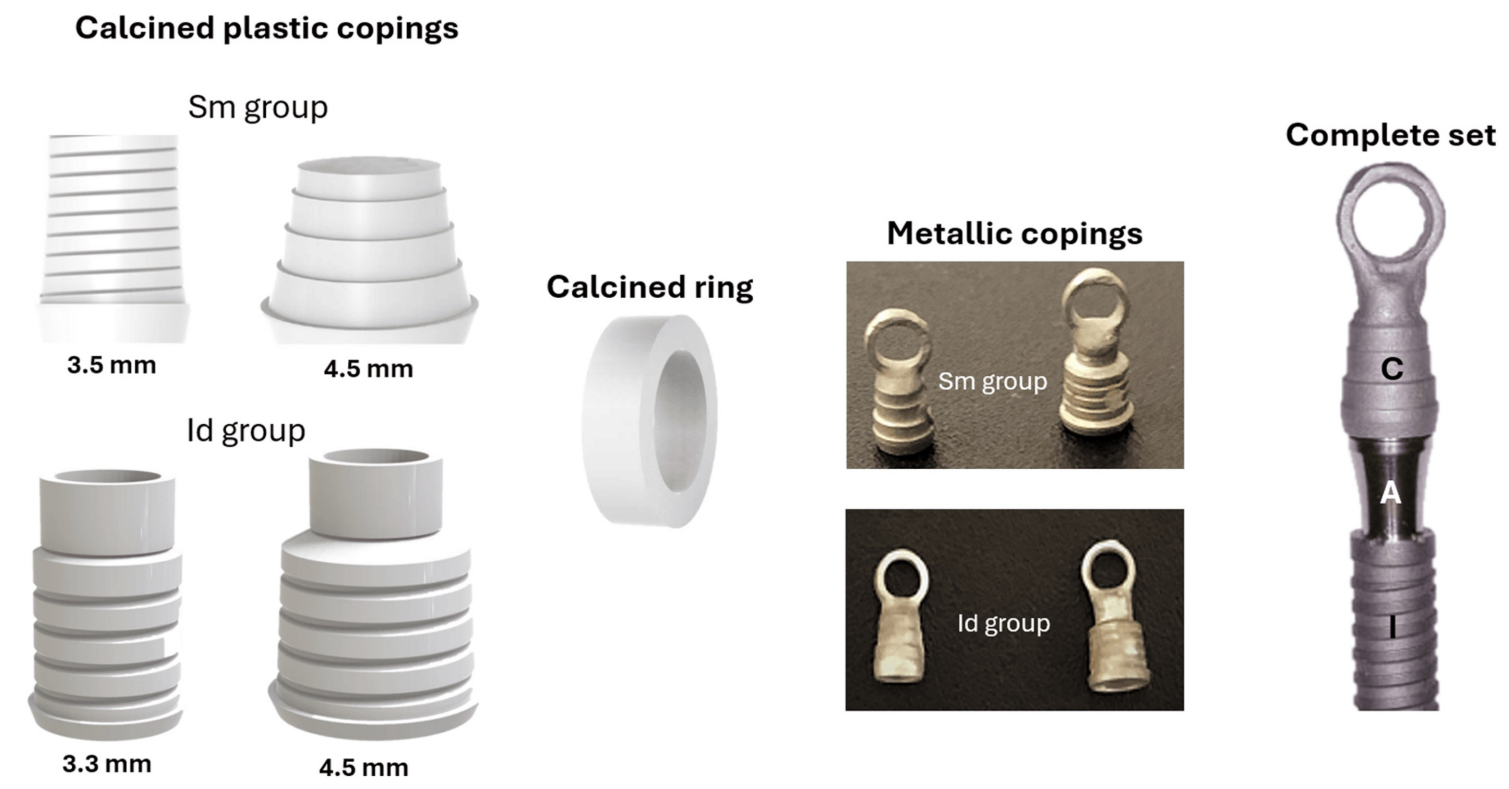
Representative image of the calcined plastic copings, calcined ring, and metallic copings obtained after the casting and the complete set I = implant; A = abutment; C = metallic coping Source: The Authors

The metallic copings were obtained from prefabricated calcined plastic copings for each abutment model. During the waxing process, a calcined ring was placed over the coping to allow a steel wire to be passed through this hole, facilitating coupling to the tensile testing equipment. Then, these sets were cast in a nickel-chromium alloy (Fit Cast Titanium, Talmax, Paraná, Brazil). After casting, each crown was blasted internally and externally with aluminum oxide (granulation 60-80 μm) to remove the coating and machined with finishing stones. These internal and external cleaning procedures to remove coating residues are the same as those used during the manufacture of conventional metal-ceramic crowns and do not aim to produce abrasion. The verification of the adaptation of each crown was performed by visual inspection, simulating the normal laboratory and clinical procedures. Figure 3 shows the calcined plastic coping, the calcined ring, the metallic copings obtained after the casting, and the complete set (implant, abutment, and metallic coping).

Sample preparation for testing

Initially, 40 Maestro Morse taper implants of 4.0 mm in diameter and 11.0 mm in length were fixed in a rigid epoxy resin G4 (Polipox, São Paulo, Brazil). Then, the abutments of each group were installed and torqued with 30 Ncm, according to the manufacturer's recommendations. Then, the cementation of the metal copings was performed using zinc phosphate cement (SS White, Rio de Janeiro, Brazil). The cement was handled according to the manufacturer's instructions, in specific proportions: a small measure of powder to four drops of liquid, mixed until a homogeneous consistency was achieved. For definitive cementation, the copings were positioned over the abutments and a controlled and constant load of 6 kgf was applied for 5 min [10], using a load cell (Industrial Técnica Oswaldo Filizola Ltda, São Paulo, Brazil), remaining after this period in the water at 37 ± 2 °C until the test is performed.

Tensile test

**Figure 4:**
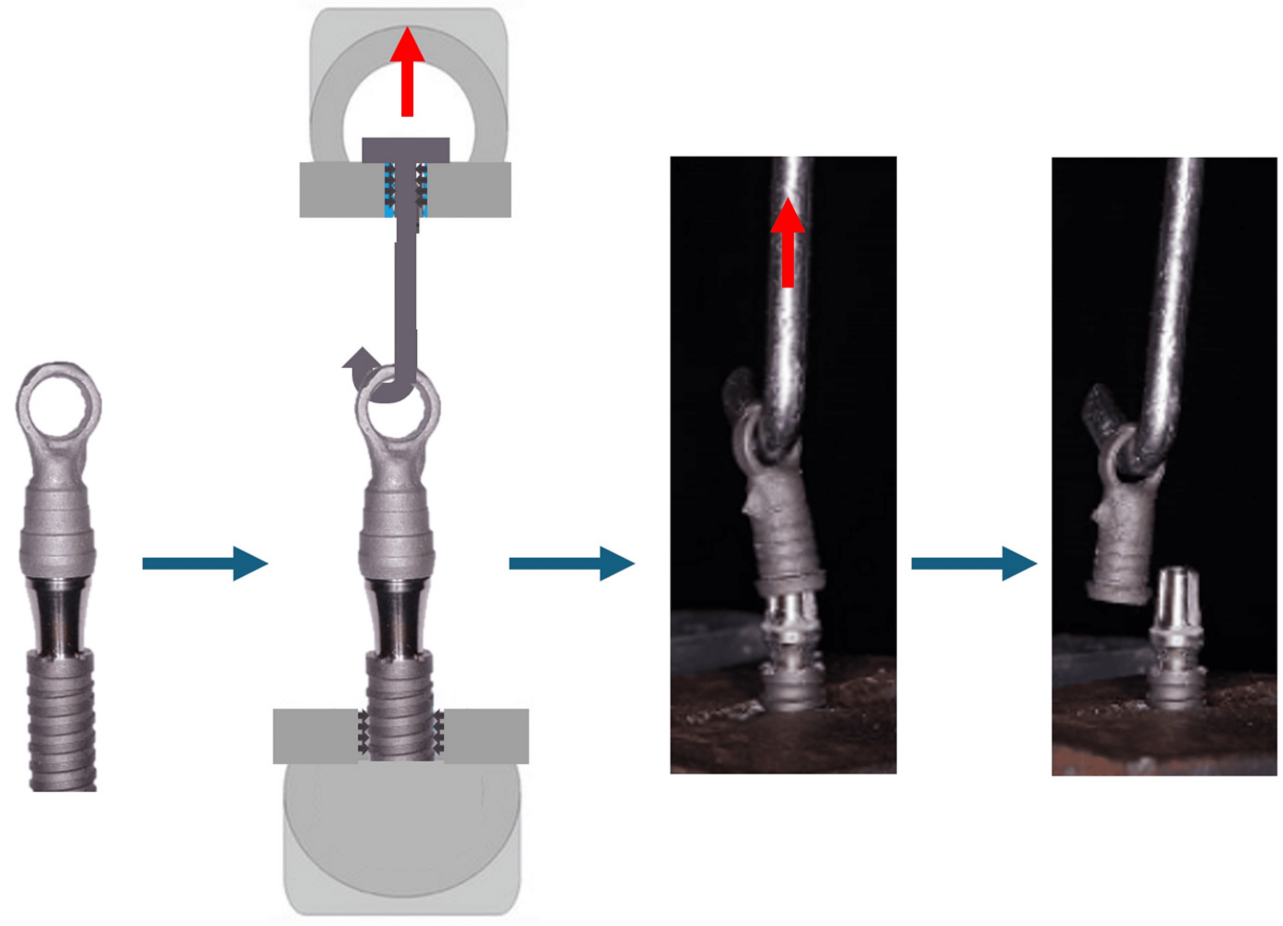
Schematic image demonstrating the sequence of preparation and execution of the tensile test Source: The Authors

The IA sets were manually fixed to the base of the test device and correctly aligned, and a metal hook was connected to the coping. The samples were then subjected to uniaxial tensile testing for coping removal using the AME-5KN universal testing machine (Industrial Técnica Oswaldo Filizola Ltda, São Paulo, Brazil), configured with a tensile speed of 3 mm/min and equipped with a displacement measurement system with a resolution of 0.001 mm. The operator conducting the test was blinded to the type of abutment used in each sample to reduce potential bias. The maximum tensile value was recorded for analysis. Figure 4 schematically demonstrates this sequence of preparation and execution of the test.

Statistical analysis

The data were analyzed using GraphPad Prism software version 5.01 (GraphPad Software Inc., San Diego, CA, USA). The Shapiro-Wilk test was applied to assess the normality of the data distribution in each group, given the small sample size (n = 10). As all groups followed a normal distribution (p > 0.05), parametric tests were deemed appropriate. Descriptive statistics were calculated for each group, including the mean, standard deviation, and standard error of the mean. Differences in tensile strength between groups were analyzed using a one-way analysis of variance (ANOVA) followed by Bonferroni’s multiple comparison post-hoc test. A significant level of 5% (p < 0.05) was adopted for all statistical analyses.

A post hoc power analysis was performed using G*Power software (version 3.1.9.7) to assess the adequacy of the sample size for detecting differences between the four experimental groups using one-way ANOVA. Based on the observed effect size (f = 0.69), an alpha level of 0.05, and a total sample size of 40 (n = 10 per group), the achieved statistical power (1 - β) was calculated to be 0.949 (94.9%). This result indicates a high probability of correctly detecting significant differences among groups, suggesting that the sample size used in this study was adequate and statistically robust for the proposed analysis.

## Results

Data normality was assessed using the Shapiro-Wilk test, which is considered the most appropriate for small samples (n = 10 per group). The results indicated that all four groups followed a normal distribution, with p-values above the significance level of 0.05: Group Sm1 (p = 0.0516), Group Id1 (p = 0.4192), Group Sm2 (p = 0.6638), and Group Id2 (p = 0.1683). Therefore, there was no statistical evidence of a violation of the normality assumption.

**Figure 5:**
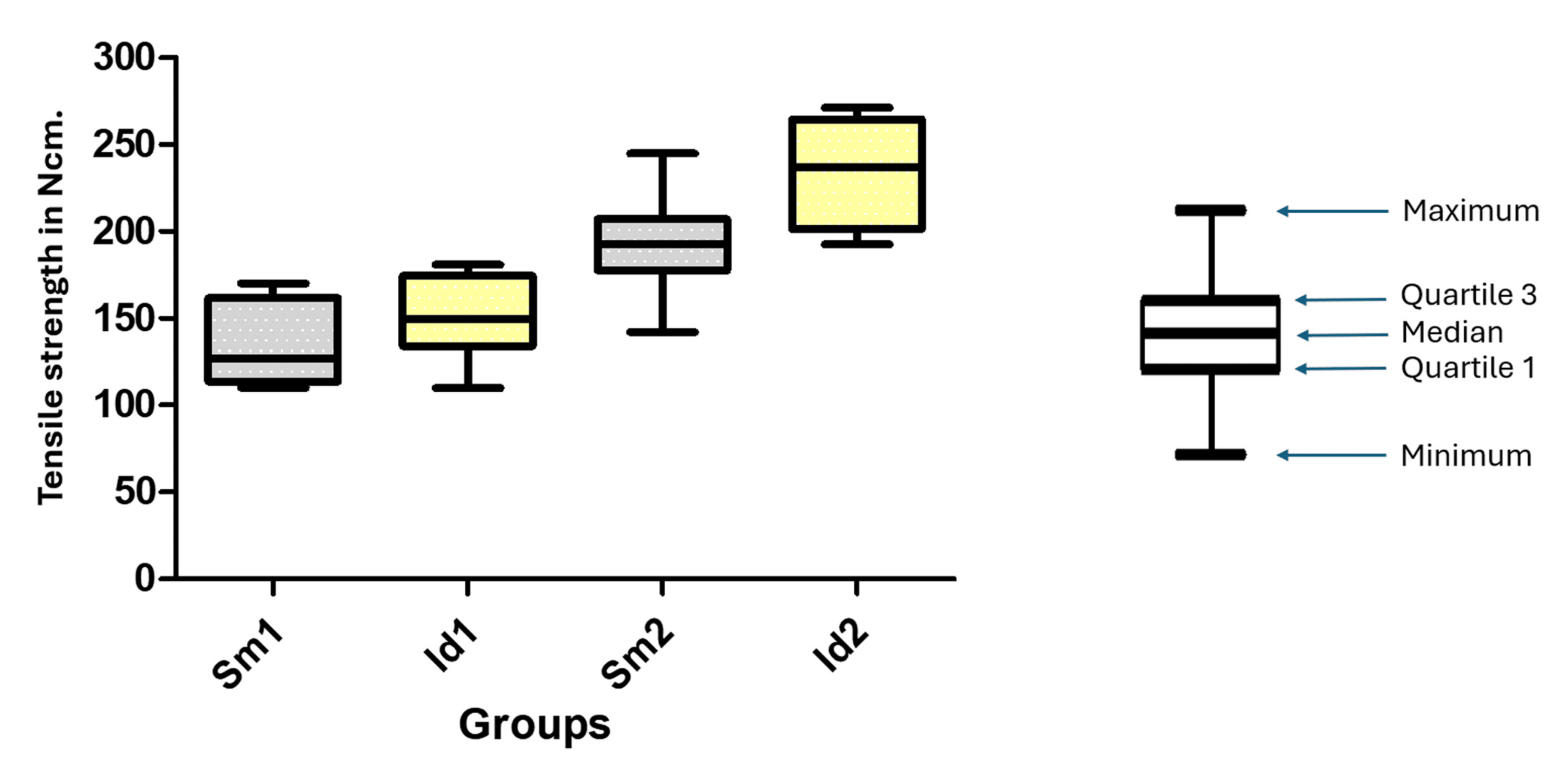
Box plot graph showing the distribution of values to remove the metalic coping in the tensile strength for each group ANOVA test p-value = 0.8696 and F-statistic = 26.66 Ncm: Newton centimeter; ANOVA: analysis of variance

Regarding the tensile strength required for crown removal across the four tested abutment models, the mean ± standard error values were as follows: Sm1 = 137.0 ± 7.666 Ncm, Id1 = 150.8 ± 7.745 Ncm, Sm2 = 191.4 ± 8.870 Ncm, and Id2 = 235.1 ± 9.785 Ncm. Figure 5 presents a box plot showing the distribution of values for each group.

Table 2 presents the results of Bonferroni’s multiple comparison test between all groups.

**Table 2:** Data comparison using Bonferroni’s multiple comparison test between all groups Values in Newton centimeter Mean Diff.: mean of difference; CI of Diff.: Confidence Interval of difference; ns.: not significant; **: significant difference; ***: highly significant difference

Comparison Test	Mean Diff.	t	p-value	Summary	95% CI of Diff.
Sm1 vs Id1	-13.84	1.143	0.2263	ns	-47.64 to 19.96
Sm1 vs Sm2	-54.37	4.491	0.0007	***	-88.17 to -20.57
Sm1 vs Id2	-98.16	8.107	< 0.0001	***	-132.0 to -64.36
Id1 vs Sm2	-40.53	3.347	0.0015	**	-74.33 to -6.726
Id1 vs Id2	-84.32	6.964	< 0.0001	***	-118.1 to -50.52
Sm2 vs Id2	-43.79	3.617	0.0089	**	-77.59 to -9.986

## Discussion

The present study aimed to evaluate the tensile strength required for crown removal in two types of prosthetic abutments -Smart and Ideale - of different diameters but identical height, to understand how variations in abutment geometry influence the retention of cemented restorations. The results demonstrated that Ideale abutments, especially the Id2 group (4.5 mm diameter), showed significantly higher tensile strength values when compared to the Smart abutments of the same diameter (Sm2). This finding supports the hypothesis that the smaller angulation of the axial walls in the Ideale design (5° vs. 11.42° in the Smart abutments) contributes to better mechanical interlocking and greater retention of the prosthetic crowns. This is consistent with the literature, which indicates that a decrease in convergence angle, within an optimal range, can enhance the retention of cemented crowns due to the increased frictional resistance along the axial walls [9,10].

Statistical comparisons confirmed significant differences between multiple groups. Notably, the Sm2 and Id2 groups exhibited the highest retention values, while the Sm1 and Id1 groups (3.5- and 3.3-mm diameter, respectively) presented the lowest among abutments. This indicates that, in addition to angulation, abutment diameter also plays an important role in the tensile resistance of cemented prostheses. Larger diameters provide a greater surface area for cement adhesion, resulting in higher retention, as shown in both Smart and Ideale models. These findings align with previous studies that reported a direct correlation between abutment geometry and cemented crown retention [6,13].

Interestingly, despite the existing diameter difference between Sm1 (3.5 mm diameter) and Id1 (3.3 mm diameter), the advantage of the Ideale abutment angulation proved to be sufficient to produce a mechanical gain, despite not presenting a statistically significant difference. This reinforces the idea that axial angulation must be considered collectively when selecting abutments, especially in clinical scenarios where space limitations or esthetic concerns exist.

Moreover, the use of zinc phosphate cement, a traditional luting agent with well-documented mechanical properties, ensured consistent and reliable bonding across all groups. This cement, widely used in clinical practice, offers sufficient strength to resist functional forces and has a long history of successful clinical application [9,10]. Although temporary cements are often recommended for their retrievability in clinical cases, zinc phosphate remains a viable option for cases that require high retention and long-term stability. Cement selection, therefore, must be individualized based on the clinical situation, including desired retrievability, occlusal load, and peri-implant tissue conditions [14,15].

Several authors have emphasized that clinical decisions regarding implant-supported restorations must consider not only implant selection but also the design of the abutment and the type of cement used [16,17]. Additionally, prosthetic success is not limited to retention values alone, i.e., the passive fit is a critical factor in preventing biological and mechanical complications in both single crowns and full-arch rehabilitations [18,19]. Unlike natural teeth, implants lack a periodontal ligament, making passive adaptation even more essential to minimize stress concentrations and maintain the integrity of the bone-implant interface.

Although this study standardized the abutment height at 4 mm, a height considered critical for retention, it is important to acknowledge that abutment height is another key factor in prosthetic stability, as demonstrated by previous authors [10,20]. However, future studies should explore the interaction between height, diameter, and angulation more comprehensively to better guide clinical decisions.

Despite its contributions, this study has limitations. As an in vitro investigation, it does not replicate the complex conditions of the oral environment, such as temperature variations, salivary flow, functional loads, and biological responses. Only one cement type and implant system were evaluated, and although the sample size was statistically appropriate, it was relatively small (n = 10 per group), which may limit generalizability. Furthermore, long-term fatigue testing was not conducted. These limitations suggest that the current findings should be interpreted with caution and confirmed by in vivo research under real clinical conditions.

Finally, while this study provides valuable insight into the mechanical behavior of cemented prostheses on different abutment geometries, the clinical selection of components must remain case-specific, taking into account not only retention but also biological and esthetic factors that contribute to long-term success.

## Conclusions

Within the limitations of this in vitro study, it can be concluded that the geometry of the prosthetic abutments significantly influences the retention of cemented crowns. Abutments with a smaller axial wall angulation (Ideale abutments) demonstrated higher tensile strength values, especially when associated with larger diameters. Therefore, Ideale abutments, particularly those with a 4.5 mm diameter, may offer better mechanical retention for cemented prostheses compared to Smart abutments of the same height. These findings highlight the importance of considering both abutment angulation and diameter when selecting components for implant-supported restorations, aiming to improve retention, stability, and the long-term success of oral rehabilitations.
